# Support Vector Machine for Lung Adenocarcinoma Staging Through Variant Pathways

**DOI:** 10.1534/g3.120.401207

**Published:** 2020-05-22

**Authors:** Feng Di, Chunxiao He, Guimei Pu, Chunyi Zhang

**Affiliations:** Department of Respiratory Medicine, Shaoxing People’s Hospital (Shaoxing Hospital, Zhejiang University School of Medicine), Shaoxing China

**Keywords:** co-expression, diagnostic model, functional pathway, lung adenocarcinoma

## Abstract

Lung adenocarcinoma (LUAD) is one of the most common malignant tumors. How to effectively diagnose LUAD at an early stage and make an accurate judgement of the occurrence and progression of LUAD are still the focus of current research. Support vector machine (SVM) is one of the most effective methods for diagnosing LUAD of different stages. The study aimed to explore the dynamic change of differentially expressed genes (DEGs) in different stages of LUAD, and to assess the risk of LUAD through DEGs enriched pathways and establish a diagnostic model based on SVM method. Based on TMN stages and gene expression profiles of 517 samples in TCGA-LUAD database, coefficient of variation (*CV*) combined with one-way analysis of variance (*ANOVA*) were used to screen out feature genes in different TMN stages after data standardization. Unsupervised clustering analysis was conducted on samples and feature genes. The feature genes were analyzed by Pearson correlation coefficient to construct a co-expression network. Fisher exact test was conducted to verify the most enriched pathways, and the variation of each pathway in different stages was analyzed. SVM networks were trained and ROC curves were drawn based on the predicted results so as to evaluate the predictive effectiveness of the SVM model. Unsupervised hierarchical clustering analysis results showed that almost all the samples in stage III/IV were clustered together, while samples in stage I/II were clustered together. The correlation of feature genes in different stages was different. In addition, with the increase of malignant degree of lung cancer, the average shortest path of the network gradually increased, while the closeness centrality gradually decreased. Finally, four feature pathways that could distinguish different stages of LUAD were obtained and the ability was tested by the SVM model with an accuracy of 91%. Functional level differences were quantified based on the expression of feature genes in lung cancer patients of different stages, so as to help the diagnosis and prediction of lung cancer. The accuracy of our model in differentiating between stage I/II and stage III/IV could reach 91%.

Lung adenocarcinoma (LUAD) is the main subtype of non-small cell lung cancer (NSCLC). In recent years, the incidence of LUAD has increased gradually in the world, and the deaths caused by LUAD accounts for nearly 50% of all lung cancer-related deaths(Kadara *et al.* 2012). Although the pathogenesis of LUAD has been well studied and certain progress has been achieved in development of therapeutic methods, LUAD remains to be one of the most aggressive and rapidly fatal tumor types, with a total survival time less than 5 years (Denisenko *et al.* 2018). The early stage of lung cancer appears no obvious clinical manifestations, and only in the advanced stage do symptoms such as chronic cough and bloody sputum gradually appear (Latimer 2018), while some early symptoms such as fatigue, shortness of breath, or upper back and chest pain are likely to be neglected. Therefore, rapid diagnosis and treatment are essential to improve the survival time of cancer sufferers (Jacobsen *et al.* 2017).

The traditional diagnostic methods for lung cancer in clinic mainly include chest X-ray and computed tomography (CT) (Prabhakar *et al.* 2018). However, in up to 25% of lung cancer cases, chest X-ray do not reveal any abnormal lesions. While the CT scan has the problem of high radiation and its accuracy also needs to be improved (Hirsch *et al.* 2017). In addition, the clinical features of early LUAD are complex, and its CT imaging is similar to that of many other diseases, like pneumonia and pulmonary infarction, which is prone to cause misdiagnosis (Pascoe *et al.* 2018). The diagnostic value for lesions <5 mm or lesions with ground glass opacity (Grade 1C) is limited. Depending on the imaging, puncture biopsy can be performed. However, this method is invasive with a risk of complications. Additionally, the approach not only cannot guarantee that tumor cells can be collected by a single puncture sampling, but also may potentially induce the risk of cancer metastasis during the invasive sampling process(Prabhakar, *et al.* 2018). Liquid biopsy, a new blood test method, can accurately detect the expression of specific genes in LUAD in a non-invasive manner. With the aid of such technique, early diagnosis of lung cancer and long-term monitoring of treatment response can be achieved according to the expression status of the specific genes in patients’ peripheral blood. Compared with traditional detection methods, gene test has high sensitivity and specificity, and can avoid the risk of cancer spreading caused by invasive testing (Hofman 2017; Pi *et al.* 2017).

The early diagnosis and detection of lung cancer are vital to improve survival, but the clinical operation is complicated, and there is no powerful diagnostic screening method available in routine practice. Delay in early diagnosis can be avoided if various barriers related to diagnosis are addressed (Cassim *et al.* 2019). The clinical staging of lung cancer is a vital process that helps to determine the treatment plan and guide the prognosis of the disease (Navani *et al.* 2015). Under different stages, the tumor microenvironment of LUAD is different to some extent. For example, with the increase of tumor stage, it is followed by increased tumor infiltrating macrophages, mast cells and neutrophils (Banat *et al.* 2015). In addition, gene expression also alters at different stages of LUAD. It has been found that the numbers of differentially expressed genes (DEGs) in IB, IIB, IIIA and IV tumors are 499, 602, 592 and 457, respectively. In early-stage tumors, DEGs are tightly related to the negative regulation of signal transduction, the apoptosis pathway and p53 signaling pathway. While in advanced tumors, DEGs are noticeably activated in transcription, response to organic substances, and biological processes associated with synapse regulation (Wang *et al.* 2017a).

The traditional method for studying disease markers relies on the expression level of single genes, as it is believed that each gene is relatively independent, which makes it possible for gene expression used in predicting the risk of illness. However, in biological individuals, genes are not relatively independent but are functionally associated. Therefore, in the study of feature gene selection across different tumor stage, finding a group of genes that are crucial for the classification of samples is the key to establish an effective classification model. Support vector machine (SVM) is a preferable method that can be used to establish the classification model. It is a new machine learning method proposed by Vapnik *et al.* based on statistical learning theory (Masoudi-Sobhanzadeh *et al.* 2019). According to the structural risk minimization principle, SVM focuses on the study of statistical learning based on small sample data, and provides a unified framework for solving the learning of limited number of samples (Van Gestel *et al.* 2001). There have been some reports on the use of SVM to classify tumors or to screen the feature genes in combination with other bioinformatics methods (Blumenthal *et al.* 2017; Kang *et al.* 2019). But this method is still in the early stage of exploration, it is important to build a more complete classification model and apply it in classification of a variety of cancers.

In order to consider the changes in patients’ molecular level and functional level from a higher dimension, this study converted gene expression information into functional imbalance variations. On the one hand, it overcame the cross-platform and cross-sample instability of a single gene marker. On the other hand, it suggested the potential pathogenic mechanism from the functional level. Meanwhile, specific genes enriched in corresponding functional pathways were likely to be important therapeutic targets or diagnostic markers in clinical practice. More importantly, our analysis found that some functional pathways only exhibited imbalance variation of functional level in a certain stage, while the imbalance did not occur in the previous stage. This suggested that the conventional treatment method excessively covers the patients, and the feature functional pathways we identified can achieve more targeted and personalized treatments. Some of the codes used to build the SVM classification model was disclosed on GitHub link: https://github.com/Zhang-Chunyi/lung-adenocarcinoma-classification-using-variant-pathways.git.

## Materials and Methods

### Data pre-processing

Gene expression files and relevant clinical data of LUAD patients were accessed from The Cancer Genome Atlas (TCGA) database (https://genome-cancer.ucsc.edu/), totally including 517 LUAD samples and expression files of 18,895 genes. The sample data were standardized as a preprocessing step. Thereafter, samples and genes with a missing value greater than 10% were eliminated, and for the remaining samples with a missing value, mean values of the corresponding genes in other samples were replaced by. According to the clinical staging characteristics, all samples were divided into 4 groups, with stage I samples the malignancy of which were the lowest as the control group. The mean value and standard deviation of each gene in the control group were calculated. Z-score normalization was then performed on all samples, and the expression of the gene in the control group was subject to a standard normal distribution with a mean of 0 and a variance of 1.

### Feature gene extraction

Coefficient of Variation (*CV*) was used to evaluate the fluctuation of genes in LUAD samples. According to the distribution of the *CV* in all genes, only 25% of the genes with the *CV* in two-tail were selected as the genes that might be related to LUAD, while the remaining 50% of the genes could be considered to be independent with LUAD due to a small fluctuation around 0. The *CV* can be calculated as shown in Equation 1:

$$CV=\frac{mean}{sd}$$ (1)

where mean refers to the average gene expression in all LUAD samples and sd refers to corresponding standard deviation. The higher the *CV* value, the more significant the positive gene fluctuation.

In order to identify the feature genes in each stage, we used analysis of variance (*ANOVA*) to assess the significance of gene expression in four groups, and *P* < 0.05 was considered statistically significant. The genes identified by *ANOVA* were deferentially expressed in at least two stages. In order to further identify the two stages, we used the TukeyHSD test algorithm to conduct pairwise comparison and finally identified the feature set of each stage.

#### Correlation analysis of the feature genes in each stage:

The interaction between genes changes with the development of LUAD. In terms of biology, two related genes have common biological functions in a healthy state, and they will cooperate or interact with each other. In terms of expression, they are co-expressed. However, in a disease state, the functions of the genes are abnormal, and the co-expression relationship is changed accordingly. Therefore, we investigated the relationship among all feature genes in different stages. Genes that had a Pearson correlation coefficient over 0.5 were considered positively correlated, but negatively correlated when the coefficient was lower than -0.5. The overlapping genes among the 4 stages were taken and then subjected to correlation analysis, with the results shown in heat map.

### Unsupervised clustering analysis

Pearson correlation coefficient was used to analyze the correlation between genes (Bishara and Hittner 2012), and the average linkage similarity matrix was used to construct the correlation coefficient matrix (Chen 2009). Unsupervised clustering analysis was performed on samples and genes based on hierarchical clustering analysis. Based on the results of unsupervised clustering analysis, we observed and analyzed the effect of differentiating samples of different cancer stages at the gene level. The clustering results were visualized using a heatmap.

### Co-expression network analysis

As the correlation between gene expression is different in different disease states, the specific system network of each disease stage should also reflect significantly different network characteristics. We constructed a specific network for each grade based on the co-expression relationship between genes. Due to the different disease states, the topological properties that were reflected by the co-expression networks were significantly different, which prompted that in different malignant grades, the efficiency of system network signal transmission was significantly different. Therefore, we analyzed the efficiency from four topological properties, which were Average Shortest Path (ASP), Closeness Centrality, Cluster Coefficient and Degree. If the edge of the network is missing, the co-expression relationship between genes disappears, then the ASP of the network increases, while Closeness Centrality, Cluster Coefficient and Degree decrease, leading to the reduction in efficiency of the network signal transmission. Finally, we used Degree of gene nodes in the network to evaluate the importance of genes. The higher the degree, the more genes would be affected when a gene is abnormally expressed. We converted the degree of all genes into a weight of 0-1 by using sigmoid function, and weight of the gene that was not in the network was minimum by default. Equation 2:

$$\text{sigmoid}\left(\text{degree}\right)=\frac{1}{1+e^{-degree}}$$ (2)

### Functional pathway enrichment

In order to further analyze the biological functions involved by feature genes in different disease stages from the functional level, functional enrichment analysis was conducted on feature genes in each stage. Fisher exact test was adopted, and the significant enriched pathways obtained were believed to be the biological functions regulated by these feature genes. Since the expression of these genes varied at different stages, and co-expression genes tended to participate in the same biological functions, we speculated that these pathways could help genes better distinguish samples of different stages. Meanwhile, these pathways with abnormal functions in different stages could be used to explain the mechanism of disease progression, and may contain potential drug targets or diagnostic markers.

### Score of functional pathway imbalance variation

As there were differences in feature genes in different stages, and all genes with functional relevance were concentrated in the same pathway, we adopted Equation 3 to calculate the overall deviation score of the pathway based on the expression of the feature genes enriched in the pathway in each sample. Equation 3:

$$\text{A}\left(\text{P}\right)=log_{2}(\frac{\sqrt{\sum_{i=1}^{m}\omega_{i}{\left(X_{i}-\mu_{i}\right)}^{2}}}{\sqrt{\sum_{j=1}^{n}\omega_{j}{\left(X_{j}-\mu_{j}\right)}^{2}}}$$ (3)

For function term P, A (P) is the score of functional imbalance, m is the number of up-regulated DEGs in the pathway, n is the number of down-regulated DEGs, ω expresses the gene weight in the co-expression network, Xi is the expression value of the up-regulated gene i, Xj is the expression value of the down-regulated gene j, μ represents the mean value of gene expression in the stage I samples and finally log base 2 is taken for conversion. Therefore, if A(P) = 0, it indicates the balance between up-regulated genes and down-regulated genes in the function. If A(P) > 0, it indicates that the up-regulated genes are dominant and the functions are up-regulated, while A(P) < 0 indicates that downregulated genes are dominant and the functions are down-regulated. Equation 1 was used to calculate the deviation degree of pathway P from the normal state.

### Recursive feature elimination (RFE)

RFE (Liu *et al.* 2015) method was adopted to screen the optimal feature sets. With the aid of RFE method, all features were randomly constituted into several small feature sets. The training set was tested iteratively by using RFE combined with cross validation, and k insignificant features were eliminated from the training set each time. Keep the cycle going until the best prediction accuracy was ensured.

### Classification model was established based on variant pathways

In order to distinguish LUAD samples in four groups of different stages by using the variant pathways with functional imbalance, we used SVM to construct a diagnostic classification model. The initialization parameters of the model included the Gaussian RBF kernel function with gamma of 0, and other parameters by default. The gridsearch was used to optimize parameters, and the optimal parameter combination was solved. The ROC curve was drawn by fivefold cross validation to evaluate the classification efficiency of the model.

### Data availability

The data used to support the findings of this study are available from The Cancer Genome Atlas (TCGA) database (https://genome-cancer.ucsc.edu/). The source code can be found at https://github.com/Zhang-Chunyi/lung-adenocarcinoma-classification-using-variant-pathways.git. 

## Results

### Data standardization

**Figure 1:**
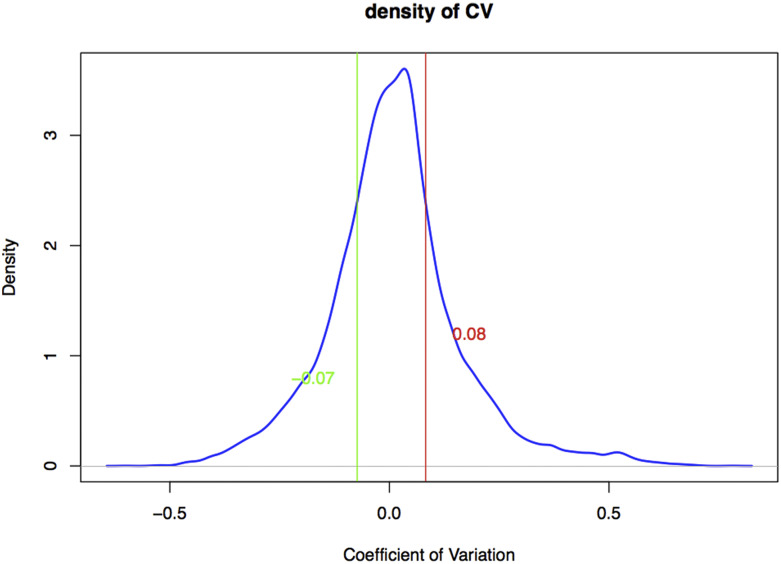
The distribution of *CV* in genes. The x axis is *CV* and the *y* axis is the distribution of density. The red and green vertical lines represent 75% and 25% of the quantile, respectively. Therefore, genes with *CV* greater than 0.08 or less than -0.07 are considered to have greater abnormal expression in LUAD.

Combined with clinical information, there were 508 LUAD samples with clear staging information, which included 277 stage I samples, 122 stage II samples, 84 stage III samples and 25 stage IV samples. After standardized treatment, 25% of the genes in two-tail were screened out according to the gene *CV*. As shown in Figure 1, genes with *CV* > 0.08 or *CV* <-0.07 were selected as candidate feature genes, and a total of 9,449 genes were screened out in the four groups. After Z-score standardization, the matrix (9449*508) data were finally obtained.

### Feature gene identification

**Figure 2:**
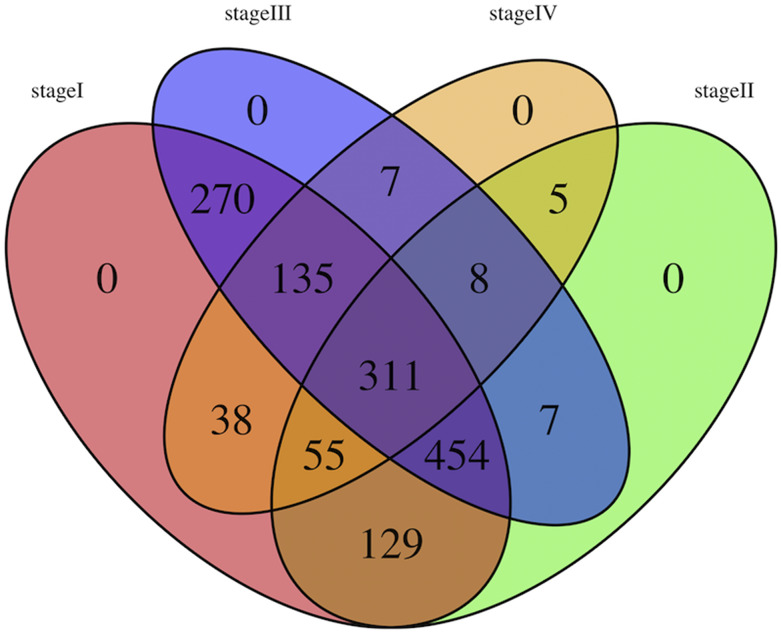
Venn diagram of four stage feature genes The four stages in the figure are marked with four colors. The intersection of any two stages represents the significant difference between the shared genes in the two stage samples.

We divided the samples into four groups: stage I, stage II, stage III and stage IV. The malignancy of LUAD increased with the increase in the sample stage. After standardization, 9,449 genes corresponding to 508 LUAD samples were obtained. In order to identify the feature genes in each stage, we first identified 1,639 genes with significant differential expression in four groups by *ANOVA*. Then we further compared the genes between groups, and finally identified feature genes in each group. In the end, 1,392 feature genes in stage I, 969 genes in stage II, 1,192 genes in stage III and 560 genes in stage IV samples were obtained. The relationship among the genes in four stages was exhibited in Figure 2, and it was found that 311 genes were shared by four stages, which indicated that the expression of these genes was different in four stages. During the malignant progression of LUAD, the expression of these 311 genes displayed a dynamic change. On the one hand, this expression pattern could be used as a clinical indicator to monitor the cancer progression of patients. On the other hand, the functions regulated by these genes were likely to be associated with cancer progression.

### Unsupervised clustering analysis

**Figure 3:**
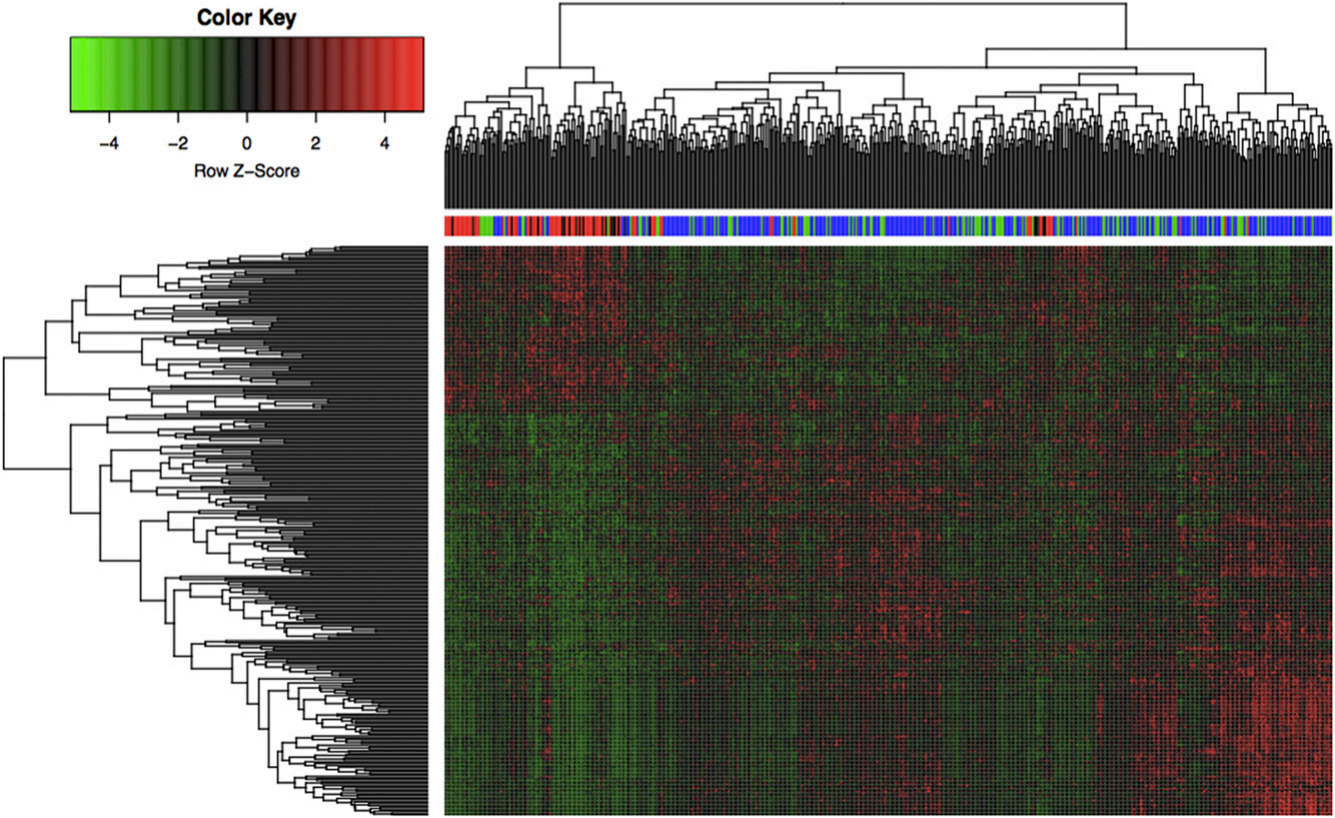
311 genes are used for unsupervised clustering analysis of four lung cancer stages The *x* axis represents the samples and the *y* axis represents the genes. Four colors are used to mark the LUAD samples with different cancer stages, blue for stage I group, green for stage II group, red for stage III group, and black for stage IV group. The red blocks represent up-regulated genes and green blocks represent down-regulated genes.

The 311 feature genes all showed differential expression in four stages, which is an important feature for monitoring disease progression. Therefore, clustering analysis was performed based on these 311 genes in four groups. Pearson correlation coefficient was used to construct a correlation matrix. Unsupervised hierarchical clustering analysis was performed to investigate the efficiency of these genes in distinguishing samples in different stages. It could be observed intuitively from Figure 3 that almost all stage III and stage IV samples were clustered together, while stage I and stage II samples were clustered together. Therefore, it could be concluded that there were significant differences between the samples with different malignant degrees at the molecular level. A high similarity could be seen in cancers of early stages (stage I- II), while a high similarity could also be seen in cancers of advanced stages (stage III- IV). It was also found that the down-regulated genes were dominant in the advanced LUAD samples (stage III- IV), while the up-regulated genes were dominant in the early LUAD samples (stage I- II), suggesting that the expression of more genes was inhibited and functional level was down-regulated in the progression of lung cancer. We also found that a certain percentage of advanced LUAD samples were clustered with early LUAD samples. This indicated that in clinical practice, the molecular level of some advanced samples was still close to that of early samples, and these samples may have a better prognosis.

### Correlation analysis

Genes with consistent function often show a significant co-expression correlation that can be divided into synergy, antagonism and compensation. In addition to the interaction between genes, the co-expression correlation is also affected by the regulatory effects of other small molecules, such as miRNAs and ceRNAs. In cancer researches, the co-expression correlation is even more important because it changes dynamically with the progress of cancer. The dynamic change provides the basis for the pathological mechanism of cancer progression, and is an important feature for dynamic monitoring patients’ medical conditions.

**Figure 4:**
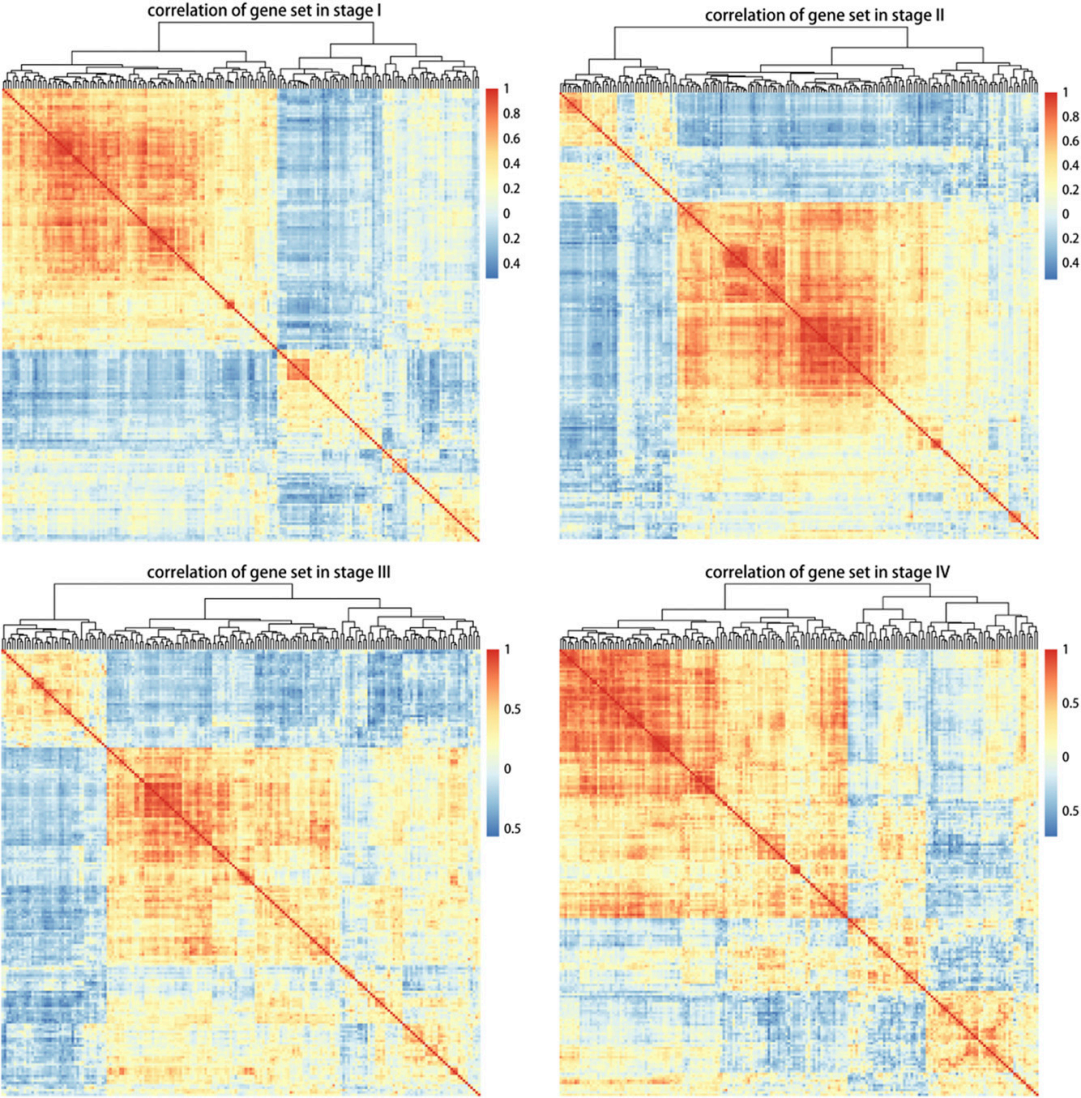
Correlation analysis of feature genes in 4 stages Each color block corresponds to the correlation coefficient of two genes, red for positive correlation while blue for negative correlation.

The correlation coefficient between any two genes in each stage feature gene set was calculated by Pearson algorithm. The number of correlated gene pairs in four stage feature sets was listed in Table 1. Most of the gene pairs were positively correlated while a few gene pairs were negatively correlated. Meanwhile, there were 2,559 overlapped gene pairs with stable expression correlation in all four stage feature sets, which involved 191 genes. It could be observed that the 191 genes were differentially expressed in four stages (Figure 4). Most genes were still positively correlated, but the correlation degree and type between any two genes varied in different stages. These results suggested that the co-expression correlation between two genes changed with the progression of LUAD.

**Table 1:** The correlated gene pairs in four tumor stages were analyzed by Pearson coefficient analysis

	Positive	Negative	Total
Stage I	14500	80	14580
Stage II	6376	28	6404
Stage III	12357	61	12418
Stage IV	6022	10	6032
Overlap	2558	1	2559

### Co-expression network analysis

**Figure 5:**
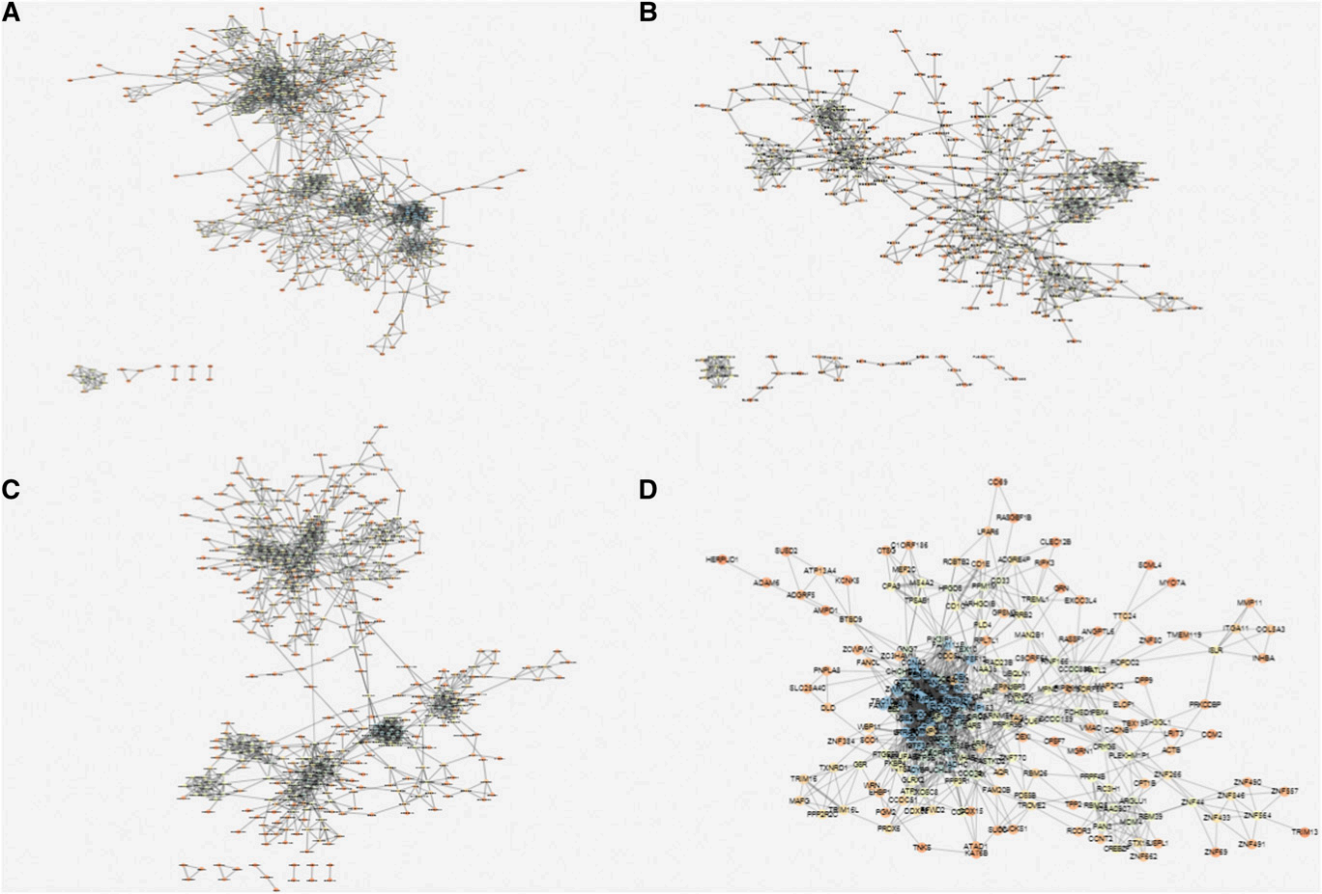
Co-expression network diagram of four stage feature genes A to D corresponds stage I, stage II, stage III, stage IV group, respectively. The closer the node color is to blue, the higher the node degree is in the network. The closer the node color is to red, the lower the node degree is. Edges between nodes represent the correlation coefficient, and the stronger the correlation, the thicker the edge.

**Figure 6:**
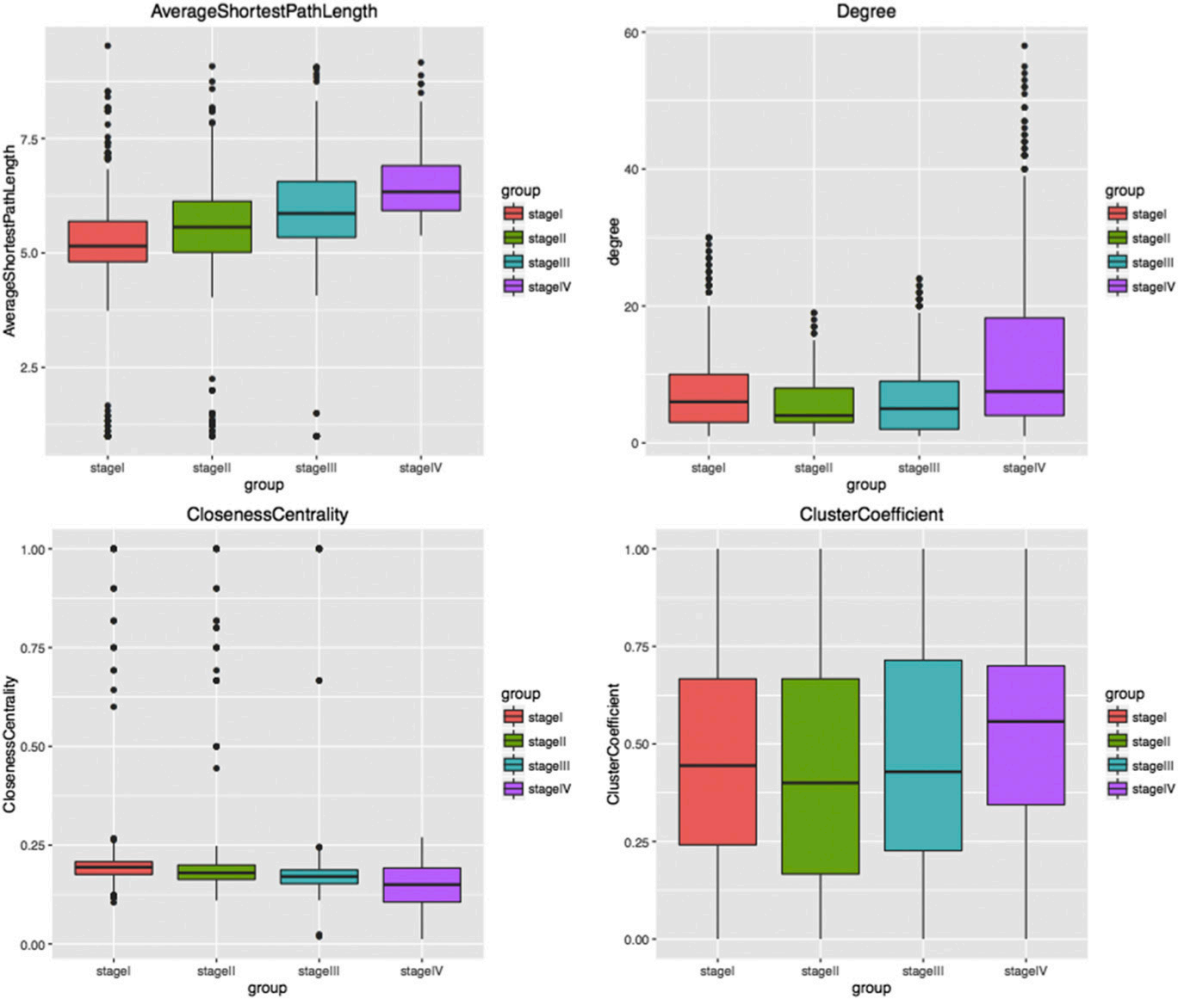
Analysis of network topological properties of four stages Analysis of 4 network topological properties, including ASP, Degree, Closeness Centrality and Cluster Coefficient. ASP measures the average state of the shortest path of a gene to other nodes in the network. Therefore, the shorter the ASP is, the more convergent the network is and the higher the signal transmission efficiency is. Degree measures the number of adjacent nodes connected by a gene in the network. Higher degree indicates that more adjacent nodes can be affected by the gene and the signal transmission efficiency is higher. Closeness Centrality reflects the degree of proximity between one node and other nodes in the network. The smaller the Closeness Centrality is, the stronger the network contractility and the closer the distance between the genes are. Cluster Coefficient represents the ability of adjacent nodes in a graph to form a complete graph. There may be submodules such as connected branches in the network with high Cluster Coefficient.

Co-expression networks were constructed based on the four stage feature sets, with genes as nodes and co-expression correlations as edges. If the two genes are positively correlated, the edge is red. If they are negatively correlated, the edge is green. The network construction was implemented by cytoscape software (Figure 5), and network analysis plug-in was used to analyze network topological properties (Figure 6). As shown in Figure 5, some genes were clearly observed to cluster in the co-expression network of each stage. Genes in each cluster were significantly co-expressed, suggesting that the genes might have consistent functions (Figure 5). Additionally, with the increase in the malignant degree of lung cancer, the ASP of the network gradually increased, indicating that the network was looser and the signal transmission efficiency gradually decreased. Similarly, Closeness Centrality decreased with disease progression, suggesting a decreased efficiency of the network. However, there was no significant difference in the degree distribution and clustering coefficient among the four stages, which were only slightly higher in stage IV than those in the other three stages.

### Functional pathway enrichment analysis

**Figure 7:**
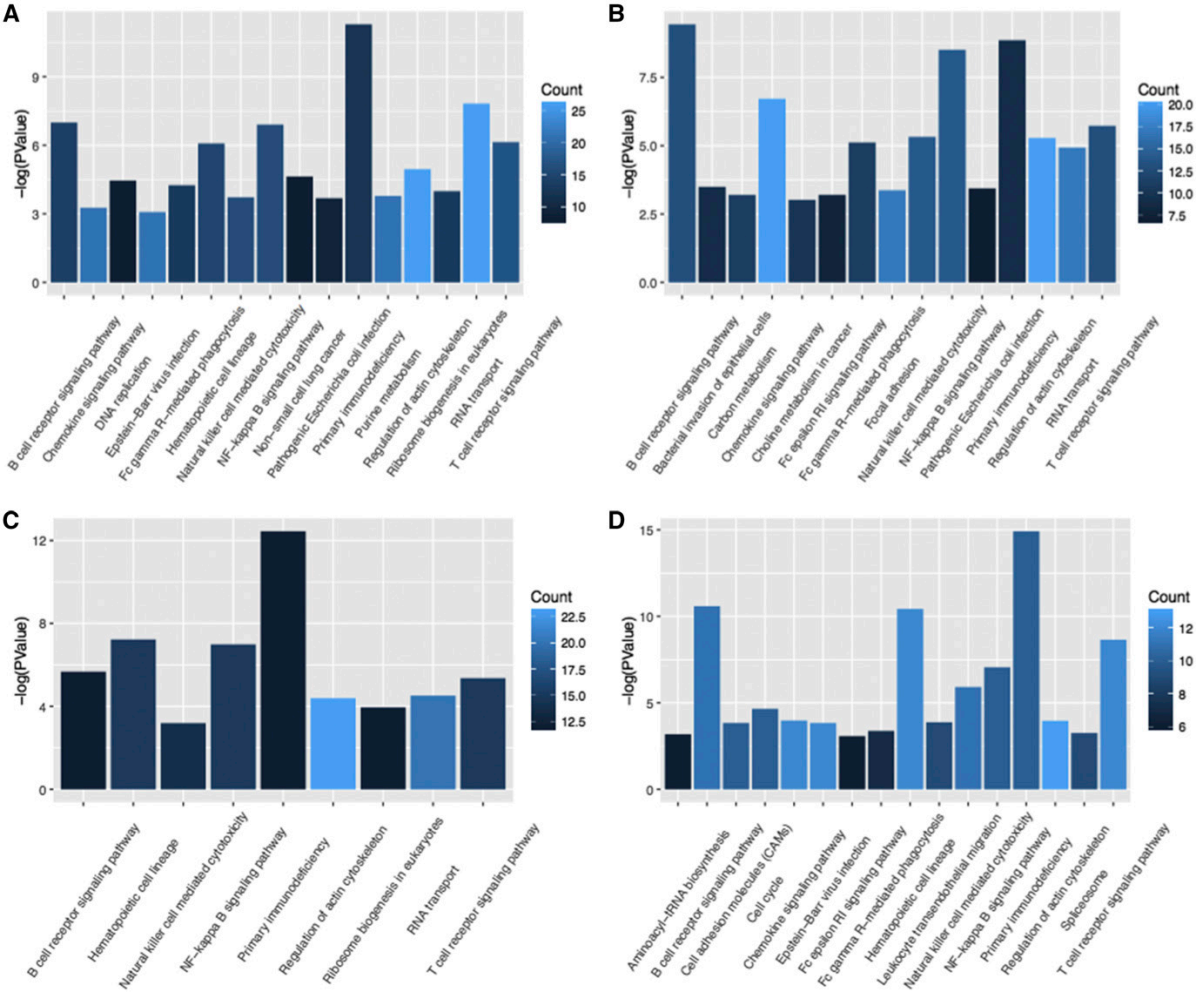
GO enrichment analysis of feature genes in four stages A-D corresponds to the pathway enrichment results of stage I-IV, respectively. The X-axis is the pathway term, and the Y-axis is the *p* value of the negative logarithmic transformation. We labeled the number of genes enriched in the pathway by dark blue and light blue. The brighter the color is, the more genes are enriched in the pathway, and darker color indicates fewer enriched genes.

Functional enrichment analysis was performed on feature genes in each stage. Fisher exact test was performed to verify the enrichment with *P* < 0.05 set as the threshold. We calculated the significant *p* value corresponding to the pathways involved in each stage and the number of genes enriched in the corresponding pathways, as shown in Figure 7. GO analysis results showed that the functions involved in stage I genes included pathogenic *Escherichia coli* infection and ribosome biogenesis in eukaryotes. The functions associated with stage II genes were focused on T/B cell receptor signaling pathway, carbon metabolism, Natural killer cell mediated cytotoxicity, Primary immunodeficiency and Primary immunodeficiency. While for stage III genes, Primary immunodeficiency was the function the genes predominantly activated in and for stage IV genes, the most enriched functional pathways were T/B cell receptor signaling pathway, Hematopoietic cell lineage and Primary immunodeficiency. The functional enrichment analysis suggested that the immune regulatory mechanism changed significantly during the progression of LUAD. Abnormal immune systems include innate immunity, specific adaptive immunity regulated by T/B lymphocytes, non-specific immunity regulated by natural killer, along with other infection- and inflammation-related functions. It further suggested that abnormal immune system was an important cause for LUAD progression.

### Score of functional pathway imbalance

**Figure 8:**
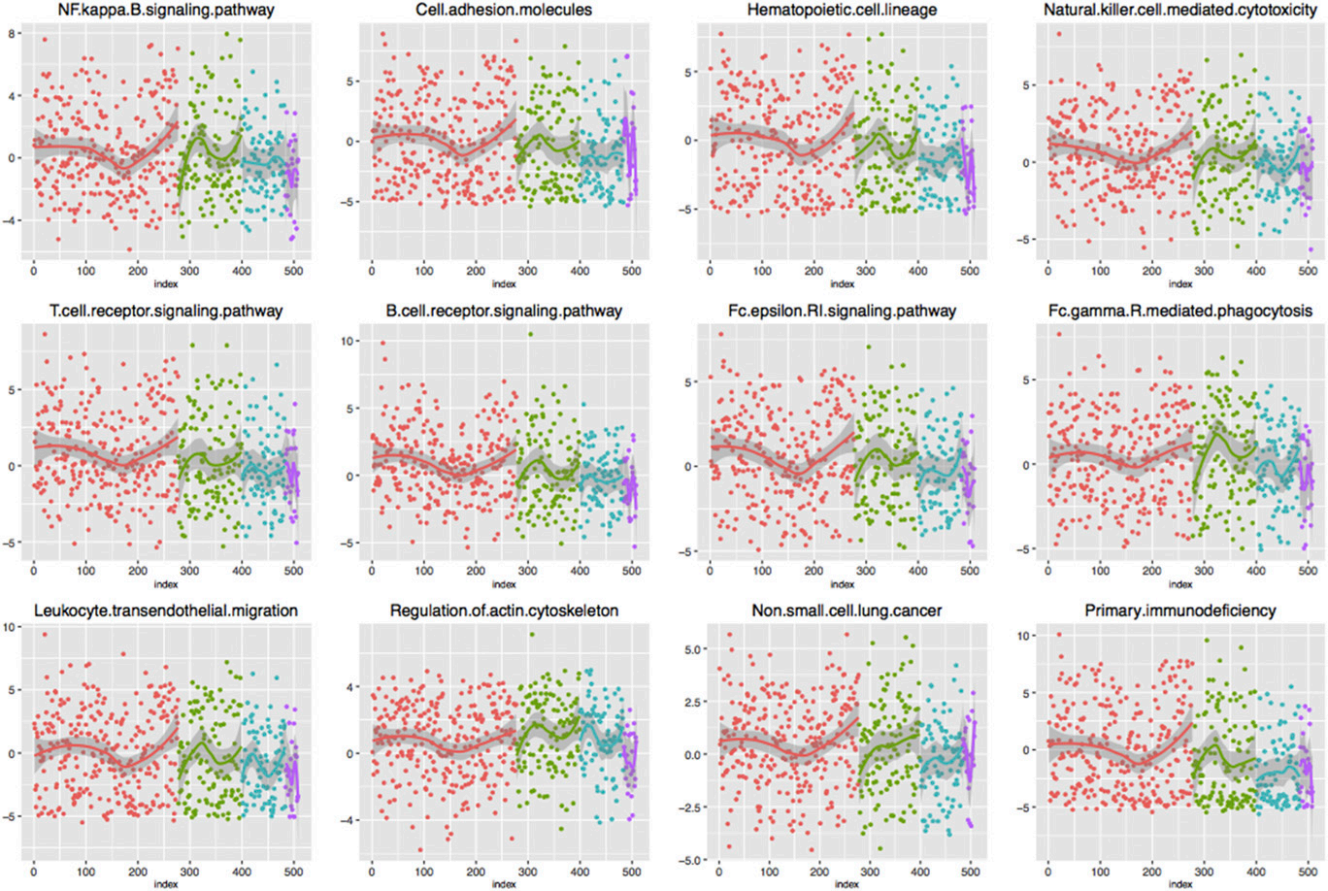
Dynamic change of 12 enriched pathways in 4 stages Stage I- IV are marked in red, green, blue, and purple respectively.

**Figure 9:**
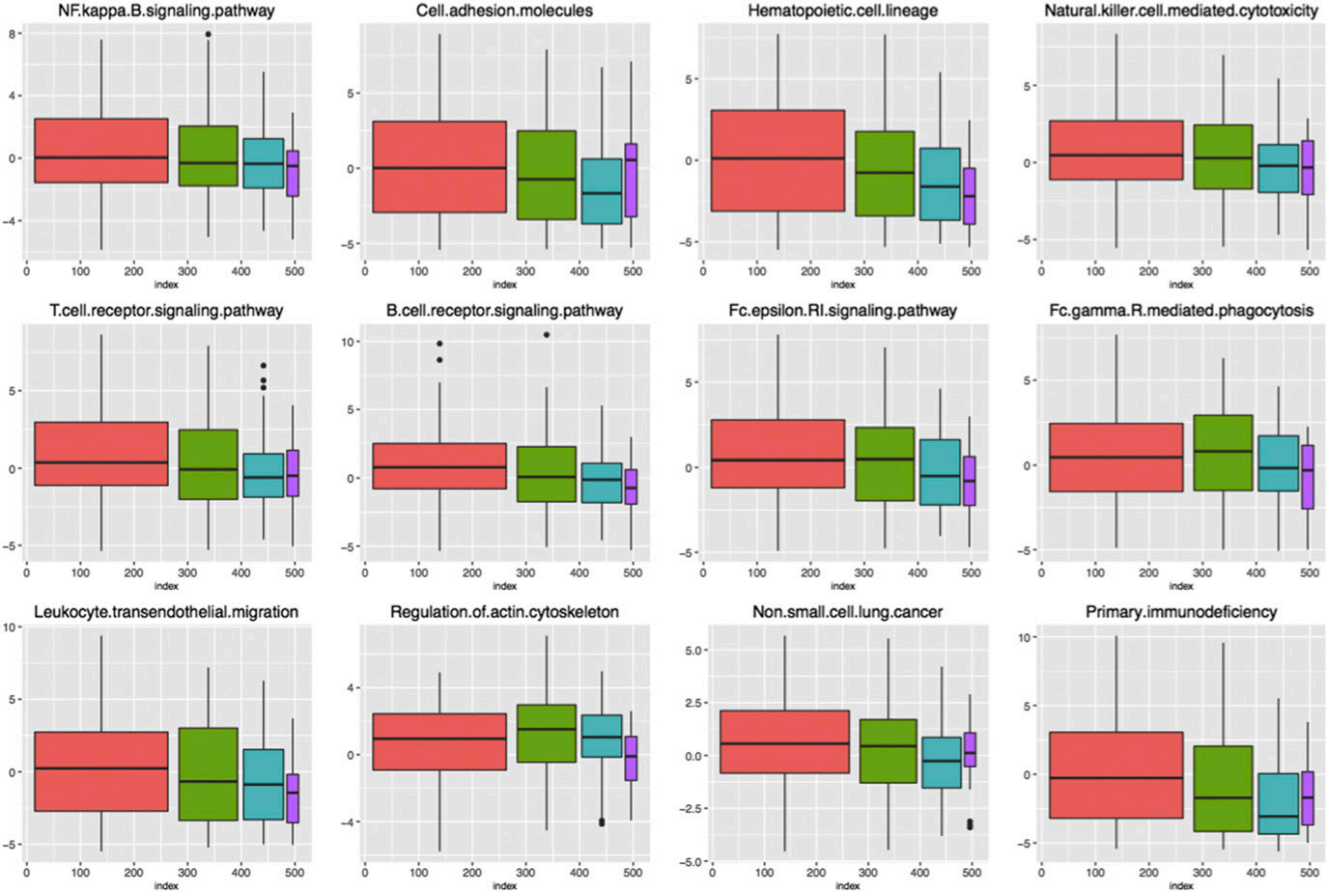
Boxplot visualizes the functional imbalance scores of 12 pathways in four stages Score boxplots of 12 pathways in stages showed the median value and confidence intervals, respectively. The four stage groups were also marked in red, green, blue and purple, respectively.

Equation 3 was used to calculate the imbalance score of each enriched functional pathway. In order to investigate whether the abnormality of these functions significantly existed in different groups, *ANOVA* was conducted to verify the imbalance score of each pathway. Finally, 12 pathways with significant differences in four stages were screened out (Table 2) (*P* < 0.05). In order to analyze the imbalance state of each pathway in the four stages more intuitively, we used scatter diagram to visualize the dynamic change of the 12 pathways (Figure 8), which could be observed using non-parametric linear fitting. It could be seen that there was no obvious change of the pathways in stage I, which fluctuated around 0. Significant fluctuations were generated from stage II. To further clarify the imbalanced variation, we compared the mean distribution of each pathway in the stages and visualized it by boxplot, as shown in Figure 9. In some pathways, the score in the four stages changed linearly, gradually increasing or decreasing. While in other pathways, the score in one or two groups was significantly different from that in the others. Therefore, it was fully confirmed that these functional pathways presented significantly different functional levels in different stages, and the classification of LUAD samples with different malignant degrees could be achieved by using these pathways.

**Table 2:** Functional significance in *ANOVA*

Pathway Term	*P* value
Primary.immunodeficiency	1.08E-05
B.cell.receptor.signaling.pathway	5.37E-05
Hematopoietic.cell.lineage	0.000310509
T.cell.receptor.signaling.pathway	0.001363854
Fc.epsilon.RI.signaling.pathway	0.002015294
Regulation.of.actin.cytoskeleton	0.004150605
Non.small.cell.lung.cancer	0.006087547
Leukocyte.transendothelial.migration	0.01039866
Cell.adhesion.molecules	0.01324628
Natural.killer.cell.mediated.cytotoxicity	0.0139198
Fc.gamma.R.mediated.phagocytosis	0.01575172
NF.kappa.B.signaling.pathway	0.01622258

### The classification model is constructed based on the pathways

**Figure 10:**
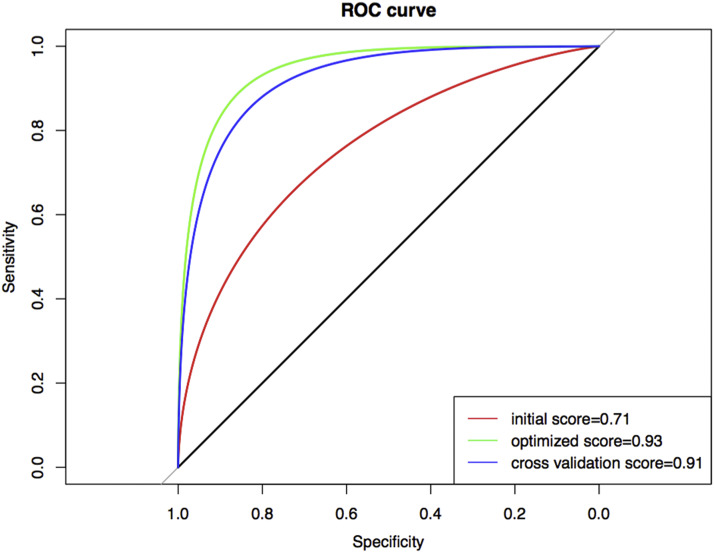
The ROC curves for accuracy evaluation of the SVM model The ROC curve evaluates the classification effectiveness of the model. The red curve is the initial model precision. The green curve is the precision of the model after feature selection and parameter optimization. The blue curve is the average precision calculated by fivefold cross validation method. In the process of cross-validation, samples are randomly shuffled each time. Four samples are taken for training and one was predicted. The X-axis represents false positive rate and the Y-axis represents true positive rate.

We used 12 pathways as features, the deviation score in each sample as the feature value, and adopted SVM to contract classification model. Since the accuracy of SVM for two-category was significantly higher than that for multi-category label classification, we combined stage I and stage II into the early benign group and stage III and stage IV into the advanced malignant group. Model training was divided into three parts including initialization, feature selection and parameter optimization. During initialization, all model parameters were set as default parameters, and the initial accuracy of the model was tested in the training set. The RFE algorithm was used to eliminate insignificant features iteratively. Four features were finally screened, including B.cell.receptor.signaling.pathway, Fc.epsilon.RI.signaling.pathway, Fc.gamma.R.mediated.phagocytosis and Regulation.of.actin.cytoskeleton. Parameter optimization was realized through the gridsearch algorithm in combination with iterative algorithm to search for the optimal parameter combination. Finally, the performance of the model was exhibited in Figure 10. The average precision of the model reached 0.91. The average precision of fivefold cross validation was close to the optimized precision of the model in the training set, which indicated that the model had not been overfitted. The predictive model could be used for prediction of early LUAD and distinguish between the benign and malignant progression.

## Discussion

Early LUAD is limited to local lesions, and some treatments such as ultrasound or X-ray and other means (Hu *et al.* 2015) are easy to cause missed diagnosis. However, in the early stages of cancer, molecular expression levels have changed (Chalela *et al.* 2017) and tumor cells undergo periodic epigenetic reprogramming to acquire new characteristics and behaviors (Wang *et al.* 2017b). In addition, the tumor microenvironment of cancer tissue lesions is often accompanied by local inflammatory response (Franzolin and Tamagnone 2019), which activates the body’s stress and immune response. In the early stage, under the regulation of innate and adaptive immunity, various genes have compensation function and fight against cancer by means of differential expression. While in the middle and advanced stage, tumor cells become dominant and malignant degree increases due to decompensation. Therefore, differential expression of genes is always present in the process of tumor occurrence and progression. Meanwhile, the expression patterns of genes are significantly different due to different pathogenesis and malignancy in different stages. Therefore, specific identification of gene expression patterns and abnormal functional levels in the four stages are of great significance for the early diagnosis of LUAD in clinical practice and the realization of personalized treatment in different stages.

In this study, we used *ANOVA* to identify the feature genes in each stage and the shared genes with different expression in the four stages. Combined with unsupervised clustering analysis, we found that 311 shared genes had significantly different expression patterns between early lung cancer (stage I/ II) and advanced lung cancer (stage III/ IV). The results revealed that these shared genes had the ability to distinguish LUAD of different malignant degrees. In order to further explore the specificity among the four stages, the feature gene set of each stage was used for subsequent analysis. Pearson correlation coefficient was used to calculate the similarity between any two genes in each stage feature gene set. Gene pairs with significant correlation were used to construct co-expression networks, which were then analyzed in the topological properties. By comparing the specific co-expression networks, it was found that the network structure changed with the increase of LUAD malignant degree, which was mentioned in the 5^th^ section of Results. The change of the network structure suggested that during the progression of lung cancer, there was dynamical change in interaction between genes under the regulation of immune system. This interaction reflected the complex change of biological system from stress compensation to decompensation in the progression of tumor. Some genes appeared correlation in the early stage. But in the process of tumor variation, the innate correlation between genes was missing, suggesting that at least one of the genes was tumor-related gene, and its expression was abnormal due to the variation. On the contrary, some genes were correlated not in the early stage, but in advanced stage, suggesting that these genes were likely to have consistent functions. However, some genes were silent in the early stage, and only when the activated genes were abnormal, the silent genes were promoted, thus creating a new correlation.

After that, we performed functional enrichment analysis on feature genes in each stage, and the results suggested that the immune regulatory mechanism *in vivo* had been significantly changed during the progression of LUAD. Abnormal immune system includes innate immunity, specific adaptive immunity regulated by T/B lymphocytes, non-specific immunity regulated by natural killer, along with other infection- and inflammation-related functions. This study further suggested that abnormal immune system was an important cause of LUAD progression. The accuracy of the diagnostic prediction model could reach 91% with the immune-related functions as features.

The innovation of this study lies in the identification of feature genes and functions of four stages of LUAD, which is of guiding significance for screening personalized diagnostic markers or therapeutic targets. Meanwhile, the fluctuation of a single gene was affected by the experimental platform and individual differences, while the functional term composed of multiple genes was relatively stable. Therefore, we built a diagnostic classification model based on gene set as the feature, which overcame the disadvantages of poor stability and low repeatability of single feature gene.

However, the limitation is that the co-expression relationship of gene dynamic changes fails to be deeply explored. The deletion and creation of co-expression relationship among genes also reflect the gene response *in vivo* with the progression of LUAD. Thus, the co-expression relationship can also be used to distinguish lung cancer of different risks. But due to the randomness and noise of gene expression fluctuations, there are many false positive results in the co-expression relationship. Meanwhile, the specific mechanism of different stages can be explained more intuitively with functions as features. Yet the detection will be more stable with stable gene pairs than using a single gene.
